# Anti-L-selectin antibody therapy does not worsen the postseptic course in a baboon model

**DOI:** 10.1186/cc3825

**Published:** 2005-11-08

**Authors:** Heinz R Redl, Ulrich Martin, Anna Khadem, Linda E Pelinka, Martijn van Griensven

**Affiliations:** 1 Professor, Director, Ludwig Boltzmann Institute for Experimental and Clinical Traumatology, Donaueschingenstrasse 13, A-1200 Vienna, Austria; 2 Managing director, La Merie S.L., Passatge Jordi Ferran, 20, E-08028 Barcelona, Spain; 3 Senior technical assistant, Ludwig Boltzmann Institute for Experimental and Clinical Traumatology, Donaueschingenstrasse 13, A-1200 Vienna, Austria; 4 Assistant professor, consultant anesthesiologist, Ludwig Boltzmann Institute for Experimental and Clinical Traumatology, Donaueschingenstrasse 13, A-1200 Vienna, Austria; 5 Professor, associate director, Ludwig Boltzmann Institute for Experimental and Clinical Traumatology, Donaueschingenstrasse 13, A-1200 Vienna, Austria

## Abstract

**Introduction:**

Anti-adhesion molecule therapy prevents leukocytes from extravasating. During exaggerated inflammation, this effect is wanted; however, during infection, blocking diapedesis may be detrimental. In this study, therefore, the potential risks of anti-L-selectin antibody therapy were evaluated in a primate model of sepsis.

**Methods:**

Sixteen baboons were anesthetized and randomized into two groups. The experimental group received 2 mg/kg of the anti-L-selectin antibody HuDREG-55 and the control group received Ringer's solution prior to the onset of a 2 h infusion of *Escherichia coli *(1–2 × 10^9 ^colony forming units (CFU)/kg body weight). Serial blood samples were drawn over a 72 h period for the measurement of tumour necrosis factor-α, IL-6 and polymorphonuclear elastase. In addition, blood gas analysis, hematology and routine clinical chemistry were determined to monitor cardiovascular status, tissue perfusion and organ function.

**Results:**

The three-day mortality rate and the mean survival time after *E. coli*-induced sepsis were similar in the two groups. The bacterial blood CFU levels were significantly higher in the placebo group than in the anti-L-selectin group. Other parameters measured throughout the 72 h experimental period, including the cardiovascular, immunologic, and hematologic responses as well as indicators of organ function and tissue perfusion, were similar in the two groups, with the exception of serum creatinine and mean arterial pressure at 32 h after *E. coli *challenge.

**Conclusion:**

Anti-L-selectin therapy did not adversely affect survival, promote organ dysfunction or result in major side effects in the baboon sepsis model. Additionally, as anti-L-selectin therapy improved the bacterial clearance rate, it appears that this therapy is not detrimental during sepsis. This is in contrast to previous studies using the baboon model, in which antibody therapy used to block CD18 increased mortality.

## Introduction

The interaction of neutrophils with endothelial cells is a key event in the host response to inflammatory stimuli. While beneficial in cases of infection, this same neutrophil-endothelial cell interaction can lead to tissue injury, especially in conditions associated with excessive inflammatory responses. L-selectin is constitutively present on leukocytes and rapidly shed upon activation. This molecule is actively involved in the early phases of neutrophil binding to the endothelium. Specifically, L-selectin initiates the initial phase of neutrophil adhesion to the endothelium, while the subsequent steps involve the β-integrins (CD11/CD18), which strengthen the adhesion of neutrophils to the endothelium and mediate the ensuing extravasation of neutrophils into tissues such as the lung [1,2]. Neutrophil products, such as reactive oxygen species and proteases, can cause tissue destruction. Thus, by inhibiting neutrophil extravasation, tissue damage could be avoided. As the first step in the process of neutrophil adhesion is mediated by the members of the selectin family (L-, E-, and P-selectin), neutrophil adhesion to the endothelium may be blocked by administration of anti-L-selectin antibodies. These anti-L-selectin antibodies could reduce organ injury by decreasing neutrophil accumulation in different organs during the inflammatory response. Animal studies support this notion, showing that the administration of neutralizing monoclonal antibodies, which recognize functional epitopes of L-selectin, reduces organ injury following ischemia-reperfusion [3], hemorrhage [4] and sepsis [5]. Our recent results in a baboon trauma model show that HuDREG-55 (a humanized monoclonal antibody specific for L-selectin) administered during post-traumatic resuscitation improves long-term survival [6].

These observations are in general agreement with previous inhibitor studies using anti-L-selectin therapies that included antibodies [3-5], as well as soluble molecules, such as low molecular weight carbohydrates like sialyl Lewis^x ^[7]. The microcirculatory protection provided by HuDREG-55 appears to be secondary to a functional blockade of the L-selectin molecule. A blockade of the L-selectin molecule is associated with three possible positive effects: decrease in L-selectin mediated polymorphonuclear granulocyte (PMN) rolling [1], prevention of L-selectin-mediated signal transduction [8], and reduction in PMN aggregation [9]. All three of these positive effects of L-selectin blockade could result in less endothelial damage due to activated PMNs. However, despite the positive effects of L-selectin blockade described in these various studies, blockade of leukocyte adhesion molecules may exhibit potential negative side effects [10], including the possibility of an increased risk of infection.

Anti-L-selectin antibody therapy interferes with the interaction of leukocytes with the endothelium at the early stage of leukocyte rolling. Thus, anti-L-selectin antibody therapy should theoretically decrease leukocyte recruitment to sites of infectious as well as non-infectious inflammation. In infectious inflammation, decreased PMN recruitment to tissue could create the risk of impaired defense in patients with bacterial or viral infections. Indeed, administration of antibodies to ICAM has been shown to increase morbidity and mortality in the baboon model of sepsis [11].

It is not known whether anti-L-selectin antibody therapy exerts similar detrimental actions. This therapy might negatively influence pathological events in sepsis by interfering with phagocyte function, thus decreasing bacterial clearance. Subsequently, this may increase organ damage and adversely affect survival. To evaluate these potential risks of anti-L-selectin antibody therapy, we tested the effect of L-selectin blockade in a non-human primate model of *Escherichia coli *sepsis. In order to simulate the worst case scenario of the trauma patient with incipient sepsis on antibody therapy, we administered the anti-L-selectin antibody just prior to the induction of *E. coli*-induced severe sepsis. We are aware, however, that the animals were not and could not be subjected to trauma prior to the induction of severe sepsis.

## Materials and methods

### Animals

Sixteen adult male Chacma baboons of the strain *Papio ursinus *weighing between 18 to 22 kg each were used in the study. These healthy animals were kept in quarantine for 3 months prior to the study and fasted overnight before the experiments. The experimental protocol was approved by the Institutional Animal Care Use Committee at Biocon Research (Pretoria, South Africa) and the animals were treated according to National Institute of Health guidelines.

### Instrumentation

Animals were anesthetized with 6 to 8 mg/kg of intramuscularly injected ketamine hydrochloride (Ketalar^®^, Parke Davis Co., Ann Arbor, MI, USA), and placed in the supine position. For spontaneous respiration, a special setup of low continuous positive airway pressure (1 to 2 mmHg) respiration was used. FiO_2 _was adjusted at 0.25 ± 0.02. Anesthesia was maintained with pentobarbital (1 to 3 mg/kg/hour) using a servo-controlled mechanism based on the electroencephalogram.

A 7F Swan-Ganz catheter (Arrow, Reading, USA) was inserted through the femoral vein and advanced into the pulmonary artery. This catheter was also used to monitor temperature. A polyvinyl catheter was introduced into the right brachial artery for arterial sampling and pressure monitoring. Catheters were connected to pressure transducers coupled to Lifescope II monitors (Nikon, Kohden, Tokyo, Japan). A triple lumen catheter (Arrow, Reading, USA) was inserted into the right brachial vein for anesthesia maintenance, administration of medication and for venous blood sampling. This catheter was removed at the end of the acute study period (6 h after the start of *E. coli *infusion). Cardiac output measurements were obtained using Edwards COM-2™ (Baxter, Glendale, CA).

After placement, the catheters were connected to a recording device and baseline data reflecting the normal simian values were collected. The animals were monitored continuously for an additional 6 h, and the catheters were then placed in a subcutaneous pouch. At 10, 24, 32, 48 and 72 h after the administration of *E. coli *bacteria the animals were again anesthetized with intramuscularly injected ketamine as described above. Subsequently, the catheters were reconnected to recording devices, and cardiopulmonary variables were measured again. Ringer's solution was administered at 5 ml/kg/h at baseline, increased to 20 ml/kg/h during sepsis and further adjusted to maintain pulmonary arterial wedge pressure at or above 6 mmHg. In several animals this value could be maintained; however, some of them were too ill to be able to achieve this goal. Although this model does not include any absolute fluid loss, sepsis will cause a relative fluid depletion due to fluid shifts into third space. At the end of each study period, the catheters were disconnected and secured in the subcutaneous pouch. No anesthetics were administered during these measurement intervals, and the animals stayed awake in their cages. At the end of the study period, the animals were again anesthetized with intramuscularly injected ketamine for measurements and were then sacrificed by administration of an overdose of pentobarbital.

### Study protocol

After cardiopulmonary stability had been achieved, *E. coli *bacteria were infused according to our previously described technique [12]. Briefly, 1 to 2 × 10^9 ^colony forming units of live *E. coli *per kilogram (Hinshaw's strain B7 (086a:61, ATCC 33985)) were infused intravenously over a 2 h period. Antibiotic therapy (gentamycin 4 mg/kg) was administered at 2, 6 and then every 12 h. The animals were observed for a 72 h period.

The animals were randomly assigned to one of two experimental groups (n = 8 per group). Group 1 received a single intravenous bolus injection of 2 mg/kg anti-L-selectin antibody (2.8 mg/ml) and group 2 received 0.72 ml/kg of Ringer's solution as placebo prior to the onset of the 2 h infusion of *E. coli*. The anti-L-selectin antibody used in the study was a recombinant humanized IgG4 isotype antibody also known as HuDREG55. The placebo group received only Ringer's solution, as an adequate isotype-matched control antibody of clinical-grade quality (same species, same isotype) was not available.

### Blood sample measurements

Heparinized blood samples were drawn at 0.5, 2, 4, 6, 10 and 24 h after start of *E. coli *infusion for blood cultures. Different lines were used for drawing blood samples and infusion of the bacteria.

Serum samples were prepared from blood drawn at -0.5 (half an hour before infusion of *E. coli*), 1, 2, 6, 10, 24, 32, 48 and 72 h to determine levels of tumour necrosis factor (TNF)-α, IL-6, and PMN elastase. TNF-α levels were determined by an enzyme-linked immunosorbent assay (ELISA) method. IL-6 was determined using an immunoassay on microplates. In this assay, a mouse monoclonal antihuman IL-6 antibody (5E1) was used for coating and a rabbit polyclonal antihuman IL-6 was used as the detecting antibody (antibodies kindly provided by WA Buurman, Maastricht, the Netherlands). Recombinant human IL-6 served as standard (kindly provided by P Mayer, Novartis, Vienna, Austria). PMN-elastase was determined with an enzyme immunoassay based on the radioimmunoassay system published previously [13].

Quantitative blood cultures were collected in Roche blood culture medium (Roche, Basel, Switzerland) and further processed, as described elsewhere [14].

Further blood samples were drawn for blood gas analysis, hematology and routine clinical chemistry. Commercially available kits were used to measure alanine aminotransferase, creatinine, and total protein (Roche, Basel, Switzerland) or lactate (Boehringer Mannheim, Mannheim, Germany). A Cobas Fara centrifugal analyzer (Roche, Basel, Switzerland) was used for these measurements. Arterial blood pO_2_, pCO_2_, pH, bicarbonate, hemoglobin, and standard base excess were determined (Radiometer ABL 330, Copenhagen, Denmark). Total leukocyte, erythrocyte and platelet count, hemoglobin and hematocrit were determined using a Coulter T890 counter (Coulter Electronics Inc., Hialeah, FL, USA).

### Statistics

Data are presented as mean ± standard error. The statistical evaluation between groups was performed, if not stated otherwise, using Kruskal-Wallis. The Bonferroni-Holm correction was used for repeated application of a statistical test. Survival data are shown in a Kaplan-Meier curve, and differences were calculated using the log-rank test.

## Results

### Survival

Following a period of stabilization after surgical preparation, baseline values for cardiovascular variables were similar in both groups prior to *E. coli *infusion and the concomitant injection of the anti-L-selectin antibody or the equivalent volume of Ringer's solution.

**Figure 1:**
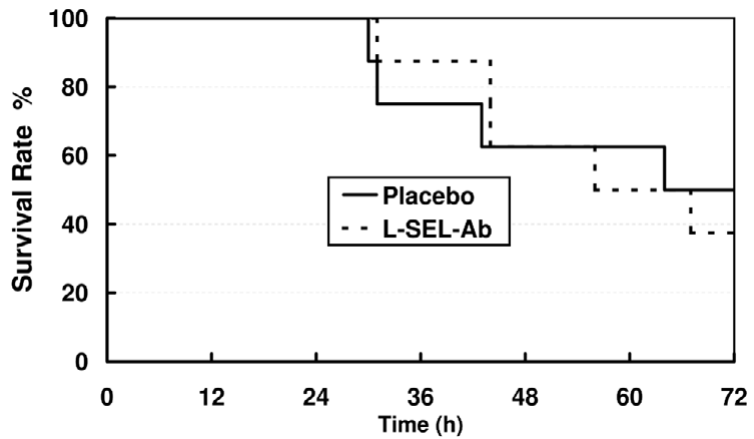
Survival rate in baboons treated with 2 mg/kg anti-L-selectin antibody (L-SEL-Ab, n = 8) or the equivalent volume dose of Ringer's solution (n = 8) with the pre-defined 72 h observation period after onset of *Escherichia coli *sepsis.

Four of eight baboons receiving Ringer's solution (placebo) died within the 72 h observation period, while five of eight animals receiving anti-L-selectin antibody treatment died (Figure 1). The mean survival time did not differ between placebo- and anti-L-selectin-treated baboons (57.3 ± 5.7 and 57.0 ± 6.7 h, respectively).

### Colony forming units

**Figure 2:**
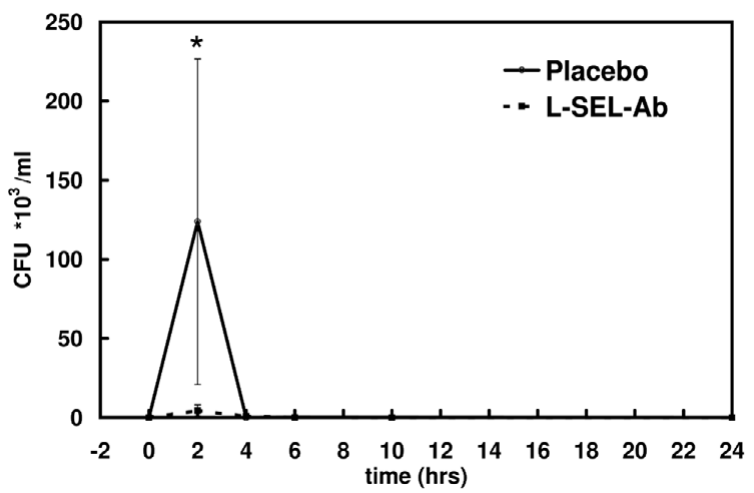
Colony forming units (CFU) in blood of baboons treated with 2 mg/kg anti-L-selectin antibody (L-SEL-Ab, n = 8) or the equivalent volume dose of Ringer's solution (n = 8) after onset of 2 h infusion (t = 0–2 h) of live *Escherichia coli*. Mean ± SE; asterisk represents *p* < 0.05.

Baboons in the placebo and in the anti-L-selectin group received the same amount of live *E. coli *(1.64 ± 0.03 and 1.61 ± 0.04 × 10^9 ^colony forming units (CFU)/kg). At the end of the 2 h infusion of *E. coli*, the CFU count in the blood was significantly higher in the placebo group than in the anti-L-selectin group (124 ± 103 versus 4.5 ± 3.6 × 10^3^/ml; *p* < 0.05) (Figure 2).

### White blood cells/elastase/erythrocytes/platelets/TNF-α/IL-6

**Figure 3:**
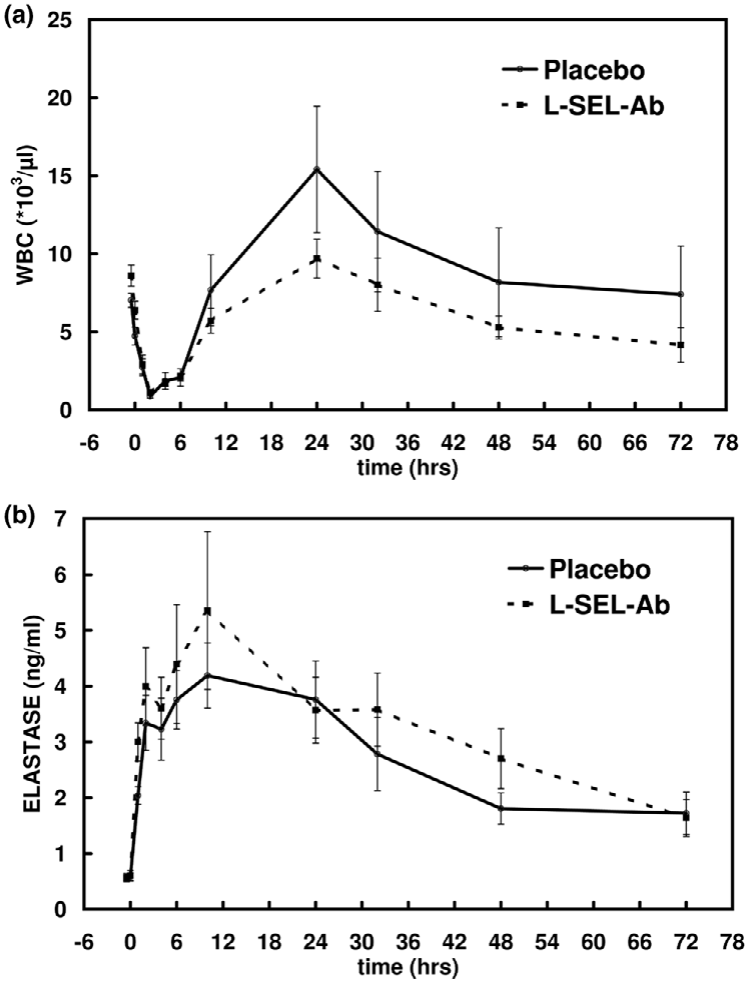
Kinetics of the inflammation parameters white blood cell count and elastase. **(a) **Time course of white blood cell (WBC) counts in baboons treated with 2 mg/kg anti-L-selectin antibody (L-SEL-Ab, n = 8) or the equivalent volume dose of Ringer's solution (n = 8) after onset of 2 h infusion (t = 0–2 h) of live *Escherichia coli*. **(b) **Time course of plasma elastase concentrations in baboons treated with 2 mg/kg L-SEL-Ab (n = 8) or the equivalent volume dose of Ringer's solution (n = 8) after onset of 2 h infusion (t = 0–2 h) of live *E. coli*.

Leukocyte counts did not differ significantly between the placebo and anti-L-selectin groups (Figure 3a), during leucopenia or during the leucocytosis period (at about 24 h). Similarly, PMN elastase in plasma, an indicator of leucocyte activation status, did not differ significantly between the groups (Figure 3b). Erythrocyte and platelet counts did not differ between the two groups either (data not shown).

TNF-α increased in both groups during the infusion of *E. coli *but was not significantly different between the two groups (Table 1). IL-6 increased after the onset of the *E. coli *infusion and persisted throughout the observation period, but did not differ significantly between the two groups (Table 1).

### Cardiovascular system

The time course of the various cardiovascular parameters demonstrated no major difference between the two groups regarding systemic vascular resistance (SVR). There was a drop in SVR at the end of the infusion of *E. coli *in both groups, followed by a decline at 24 h. Hemodynamic responses as well as gas exchange data, including heart rate, mean arterial pressure, cardiac output, pulmonary artery pressure, pulmonary arterial wedge pressure, peripheral vascular resistance, arterial pO_2_, and arterial pCO_2_, are summarized in Table 2. The only significant cardiovascular difference was found in mean arterial pressure at 32 h, but was not reflected in cardiac output and SVR.

Tissue perfusion, as reflected by arterial base excess and lactate, did not significantly differ between the two groups (Table 3). The trend in fluid infusion requirements was higher in the placebo group than in the antibody group, but this was not statistically significant (Table 4). Accordingly, there were no differences between the groups in hematocrit or total protein concentrations (Table 4).

### Organ function

Kidney and liver function, as reflected by blood urea nitrogen and alanine transferase, respectively, were similar in the two groups (Table 5). The anti-L-selectin group, however, showed significantly higher concentrations of serum creatinine at 48 h compared to the placebo group (*p* = 0.047). This potential evidence of kidney dysfunction was not histomorphologically confirmed. The trend in urine volume was even higher in the anti-L-selectin group, although not significant (*p* > 0.05, Table 5).

Arterial oxygen pressure and lung wet weight (14.6 ± 1.2 versus 15.1 ± 2.3 g/kg body weight) were used as indicators of pulmonary function. No significant differences were found between the placebo and anti-L-selectin group using either of these markers of lung injury.

## Discussion

Adhesion molecules play an important role in the interaction between leukocytes and the endothelium in acute inflammation in conditions such as traumatic-hypovolemic shock [4], gut ischemia-reperfusion [15], or myocardial infarction [3]. Although some studies have found beneficial effects [16] or no adverse events [17] in inflammatory conditions associated with septic foci using anti CD11/18 antibodies, there is still major concern regarding the use of anti-adhesion therapy in septic situations. A combination of anti-E/L-selectin resulted in elevations in IL-6, IL-8, and Tumour Necrosis Factor Receptor-1 (TNFR-1) when used in a septic model of heat killed *E. coli *followed by live *E. coli *[18]. On the other hand, administration of anti-L-selectin in an intravenous *E. coli *model was beneficial [19]. This group could show, however, that the route of infection is important for the efficacy of the treatment. Anti-L-selectin treatment worsened the late course of the intrabronchial *E. coli *disease [19]. A similar model using intrabronchial *E. coli *in rats showed that treatment with anti-ICAM-1 led to increased mortality [20]. Anti-CD11b treatment did not change mortality rates, although harmful effects could not be excluded [20]. These effects were dependent on the dose of the antibodies. Thus, one has to bear in mind that experimental protocol differences may be a reason for mixed published results from different studies. Though not primarily intended for use in sepsis, anti-adhesion antibodies infused for other reasons (trauma, shock, ischemia/reperfusion) could be present at the onset of septic events. Interestingly, anti-L-selectin antibody therapy in the present study did not adversely affect the 3 day mortality rate or the mean survival time, indicating that it had no overall adverse effects on the pathogenetic course of sepsis in this well established model of baboon sepsis [12]. As this is a very severe model, accelerating negative effects would have been especially expected if anti-L-selectin treatment had been detrimental. As the model is severe, however, worsening of the septic state could also be hard to detect. Thus, detrimental effects may not be completely excluded by the study. On the other hand, no beneficial effects were expected as this is a pure sepsis model. In this case, anti-adhesion molecule treatment is not an option. It could, however, be a possible treatment in a trauma situation, where increased PMN extravasation is present. Sepsis is a common complication during the post-traumatic course. Therefore, this study focused on the safety issues of anti-L-selectin during a possible post-traumatic sepsis.

In other studies, a decreased expression of cell surface L-selectin was associated with worse outcome in septic trauma patients or patients suffering from multiple trauma [21,22]. Reduced L-selectin levels lead to an increase in mortality after sepsis [23]. Seidelin *et al*. [24] proposed a cutoff point of 470 ng/ml for the level of soluble L-selectin predicting survival in septic patients. Moreover, this shedding can cause an increase in TNF-α receptors on PMNs [25] and thereby potentate the harmful actions of the PMNs. Furthermore, shedding of L-selectin serves as a signalling event for increasing the respiratory burst activity, which could exaggerate tissue damage [26]. Blocking L-selectin may, therefore, be beneficial not only by inhibiting extravasation, but also by modulating signal transduction pathways.

Studies with L-selectin knock-out mice have demonstrated a reduction in lymph node trafficking, a process that is normally required for proper generation of an immune response [27]. One may expect, therefore, that treatment with anti-L-selectin antibody would cause marked immune suppression. In contrast to these expectations, however, recent experiments have shown that mice treated with anti-L-selectin antibody can still effectively eliminate viruses [28] and parasites [29]. Administration of an anti-L-selectin antibody resulted in a reduced pulmonary injury in a sheep ischemia/reperfusion model but did not reveal any influence on neutrophil functions like respiratory burst [30]. This demonstrated a protective effect for secondary organ damage. Positively, the treatment did not inhibit the ability of PMN to kill microorganisms. The small-molecule pan-selectin-inhibitor TBC-1269 has been demonstrated to be protective against neutrophil recruitment and to improve survival rates in a two-hit model of hemorrhagic shock with additional lipopolysaccharide challenge [31]. Another study in a murine two-hit model of ischemia/reperfusion and cecal ligation and puncture used the sialyl Lewis^x ^analogue fucoidin, a sulphated polymer of L-fucose. The study revealed that fucoidin attenuates selectin-mediated neutrophil adherence but not neutrophil recruitment. Furthermore, fucoidin administration resulted in improved morphologic pathology [32]. These data could be a sign for the importance of selectins as cell signalling molecules rather than their function in adhesion.

The findings of the current study further support the potential safety of anti-L-selectin therapy in the presence of bacterial infection. Interestingly, anti-L-selectin antibody administration was associated with improved bacterial clearance. This was demonstrated by a significant reduction of the CFU count in blood in the anti-L-selectin group, although the amount of infused *E. coli*/kg body weight was virtually identical in both groups. The mechanism behind this intriguing observation is not clear. A possible role for the reticuloendothelial system and concomitant complement activation might be involved. The role of L-selectin in this respect is, however, hard to determine from our data. One could speculate that the signal transduction properties of L-selectin may play a role. These properties could enhance the activity of the reticuloendothelial system. This could enhance the clearance of the bacteria. Moreover, certain signal transduction pathways could induce complement activation. The complement system efficiently kills bacteria. Therefore, both entities could lead to an increased clearance of bacteria already during the infusion phase. This finding is in concert with the fact that anti-L-selectin did not negatively influence respiratory burst [30]. Moreover, L-selectin signalling can lead to increased bacterial killing capacity [26,33].

In the current experiment, the presence of monoclonal antibodies to L-selectin did not alter the pro-inflammatory response to septic stimuli as reflected by circulating levels of TNF-α or IL-6 measured up to 72 h after bacterial challenge. These results differ from previous studies where anti-adhesion therapy with monoclonal antibodies to E- and L-selectin has been reported to increase the release of pro-inflammatory cytokines IL-6, IL-8, and TNFR-1 in a baboon model of sepsis [18]; however, this previous study differs from the current one in its protocol. Twelve hours after the initial bacterial challenge, anti-adhesion therapy in this study was followed by the administration of killed bacteria [18], which itself causes a sepsis-like condition.

Organ-specific measurements, such as gas exchange, wet lung weight and liver transaminase levels, showed that there were no negative side effects of anti-L-selectin antibody administration. The only difference in organ specific function between placebo and anti-L-selectin antibody was found in serum creatinine levels measured at one time point only. Furthermore, neither macroscopic pathology, histopathology nor urine volume revealed any differences in renal injury or function between the groups (data not shown). Hemodynamically, the mean arterial blood pressure at 32 h, but at no other time point, was significantly lower in the anti-L-selectin group than in the placebo group. However, there were no differences in SVR or cardiac output at this time point. This resembles results found in an ovine ischemia/reperfusion model in which administration of an ovine anti-L-selectin reduced arterial blood pressure almost to normal levels [30]. Furthermore, the general cardiovascular response as well as the resuscitative fluid requirements were similar in both groups.

The lack of a negative effect of the anti-L-selectin antibody on the immunoinflammatory response in the current experiment is in concordance with *in vitro *studies using this antibody. These *in vitro *experiments showed that the anti-L-selectin antibody impaired neither PMN phagocytosis (of FITC-labeled opsonized *E. coli*) nor respiratory burst (measured by oxidation of fluorogenic substrate using flow cytometry). Similarly, the anti-L-selectin antibody did not interfere with endotoxin induced IL-1 synthesis by monocytes (all *in vitro *data on file at Scil Biomedicals, Martinsried, Germany). Moreover, *in vivo *evidence suggests that anti-L-selectin therapy even has beneficial effects in endotoxemic models. In mice lacking cell surface expression of L-selectin, death from endotoxin is largely prevented [34] and anti-L-selectin therapy prevents endotoxin-induced leukocyte sequestration [35].

In contrast to these positive reports using anti-L-selectin approaches, an antibody to CD18 has been shown to increase susceptibility to infection with *Pseudomonas aeruginosa *in rabbits [36]. The explanation for this discrepancy between therapies directed at L-selectin versus CD18 may be that CD18 is directly involved in neutrophil phagocytosis, as CD18 deficient leukocytes fail to increase phagocytic function in response to stimulation [37]. Thus, our current *in vivo *findings not only corroborate the results of *in vitro *studies showing that blockade of L-selectin did not inhibit leukocyte function, but also show that anti-L-selectin seems to improve bacterial clearance. The exact mechanism and the clinical relevance of the improved bacterial clearance after L-selectin antibody administration are not known and will, therefore, require further investigation.

## Conclusion

Anti-L-selectin (antibody) therapy did not adversely affect survival, promote organ dysfunction or result in major side effects. This fact and the improved bacterial clearance rate observed in the baboons receiving anti-L-selectin antibodies indicate that septic episodes occurring during anti-L-selectin therapy are probably not dangerous. These findings are particularly important as sepsis is a common complication in the post-traumatic and post-hemorrhage course.

## Key messages

• 	Anti-adhesion therapy with anti-L-selectin did not adversely affect survival, promote organ dysfunction or result in major side effects in a baboon live *Escherichia coli* sepsis model.

• 	Moreover, anti-L-selectin treatment improved bacterial clearance rate.

• 	Thus, anti-L-selectin therapy can be safely used in inflammatory settings such as trauma possibly without an increased risk of sepsis.

## Abbreviations

CFU = colony forming units; IL = interleukin; PMN = polymorphnuclear granulocyte; SVR = systemic vascular resistance; TNF = tumour necrosis factor.

## Competing interests

The authors declare that they have no competing interests.

## Authors' contributions

HRR performed experiments and evaluation. UM planned the study and produced HuDreg55. AK organised the study. LEP critically revised the article. MvG analysed and interpretated the data.

## Figures and Tables

**Table 1:** TNF-α and IL-6 in baboons infused with live *Escherichia coli *and treated with placebo or anti-L-selectin antibody

	Time (hours)										
	-0.5	0	1	2	4	6	10	24	32	48	72

TNF-α (pg/ml)											
Placebo	0 ± 0	2 ± 2	5,950 ± 1,762	6,325 ± 2,026	244 ± 71	55 ± 18	28 ± 15	21 ± 7	19 ± 7	8 ± 5	0 ± 0
L-SEL-Ab	6 ± 4	1 ± 1	9,048 ± 2,227	6,648 ± 1,465	236 ± 45	60 ± 9	39 ± 7	33 ± 11	34 ± 11	37 ± 15	39 ± 15
IL-6 (pg/ml)											
Placebo	4 ± 3	17 ± 8	2,059 ± 263	6,968 ± 718	7,331 ± 963	6,082 ± 832	5,364 ± 773	3,032 ± 900	1,921 ± 860	2,435 ± 1,635	896 ± 73
L-SEL-Ab	13 ± 5	6 ± 4	2,270 ± 665	7,392 ± 802	7,490 ± 773	7,075 ± 765	5,952 ± 500	3,547 ± 644	2,550 ± 689	1,684 ± 761	907 ± 77

IL-6, interleukin-6; L-SEL-Ab, anti-L-selectin antibody; TNF-α, tumor necrosis factor-α.

**Table 2:** Hemodynamic responses in baboons infused with live *Escherichia coli *and treated with placebo or anti-L-selectin antibody

	Time (hours)										
	-0.5	0	1	2	4	6	10	24	32	48	72

Heart rate (beats/minute)											
Placebo	124 ± 3	125 ± 3	156 ± 9	171 ± 7	170 ± 5	160 ± 5	141 ± 7	142 ± 6	138 ± 9	139 ± 5	151 ± 11
L-SEL-Ab	122 ± 5	121 ± 4	160 ± 9	177 ± 7	165 ± 3	168 ± 6	151 ± 5	142 ± 15	141 ± 14	143 ± 10	158 ± 10
MAP (mmHg)											
Placebo	113 ± 6	122 ± 3	102 ± 8	72 ± 5	103 ± 5	117 ± 6	95 ± 9	75 ± 11	75 ± 6	65 ± 11	70 ± 6
L-SEL-Ab	119 ± 4	122 ± 3	104 ± 6	72 ± 5	97 ± 5	111 ± 6	85 ± 10	51 ± 8	51 ± 7	61 ± 12	79 ± 8
CO (l/minute)											
Placebo	3.1 ± 0.2	3.2 ± 0.2	4.3 ± 0.4	4.6 ± 0.4	4.4 ± 0.4	4.2 ± 0.4	2.5 ± 0.2	3.5 ± 0.4	4.2 ± 0.3	3.6 ± 0.3	3.7 ± 0.2
L-SEL-Ab	3.1 ± 3.1	3.1 ± 3.1	4.8 ± 4.8	5.5 ± 5.5	4.8 ± 4.8	4.6 ± 4.6	2.5 ± 2.5	3.0 ± 3.0	3.8 ± 3.8	3.6 ± 3.6	3.8 ± 3.8
MPAP (mmHg)											
Placebo	13 ± 1	14 ± 1	14 ± 1	13 ± 1	14 ± 2	18 ± 2	17 ± 3	17 ± 2	18 ± 3	15 ± 1	16 ± 3
L-SEL-Ab	13 ± 1	13 ± 1	13 ± 1	14 ± 1	14 ± 1	18 ± 1	16 ± 1	16 ± 2	16 ± 1	16 ± 1	15 ± 3
PWP (mmHg)											
Placebo	3.5 ± 0.4	3.6 ± 0.5	4.4 ± 0.8	4.5 ± 1.0	4.8 ± 0.9	5.4 ± 0.8	1.4 ± 0.6	1.5 ± 0.5	3.5 ± 0.3	2.0 ± 0.7	1.5 ± 1.0
L-SEL-Ab	4.6 ± 0.7	4.5 ± 0.6	4.8 ± 0.6	5.5 ± 0.9	4.6 ± 0.8	4.9 ± 0.8	2.9 ± 0.6	2.8 ± 0.4	2.7 ± 0.4	3.6 ± 0.9	3.7 ± 0.3
PVR (dyn s cm^-5^)											
Placebo	264 ± 21	258 ± 16	180 ± 11	160 ± 17	180 ± 20	265 ± 41	542 ± 112	399 ± 80	285 ± 58	292 ± 22	301 ± 78
L-SEL-Ab	220 ± 20	223 ± 18	142 ± 22	124 ± 15	162 ± 21	243 ± 30	441 ± 58	362 ± 50	296 ± 27	267 ± 25	279 ± 70
arterial pO_2 _(mmHg)											
Placebo	100.4 ± 6.8	101.5 ± 6.9	97.7 ± 5.2	104.8 ± 4.5	105.0 ± 4.3	100.2 ± 4.3	88.2 ± 2.5	84.7 ± 4.6	76.7 ± 6.4	73.1 ± 7.0	63.4 ± 6.5
L-SEL-Ab	101.9 ± 5.9	102.2 ± 4.4	105.0 ± 6.3	109.9 ± 4.0	105.9 ± 5.7	103.6 ± 6.0	91.0 ± 5.7	89.1 ± 5.4	82.2 ± 3.5	88.7 ± 7.5	77.6 ± 8.3
arterial pCO_2 _(mmHg)											
Placebo	48.8 ± 1.1	46.4 ± 1.0	45.0 ± 1.2	41.1 ± 1.2	34.6 ± 2.3	36.3 ± 0.9	33.2 ± 1.1	33.9 ± 1.9	34.1 ± 2.4	40.5 ± 3.2	45.7 ± 3.3
L-SEL-Ab	43.7 ± 1.5	43.1 ± 1.5	40.9 ± 0.4	39.1 ± 0.9	35.0 ± 1.7	34.5 ± 0.9	28.9 ± 2.3	31.5 ± 2.3	33.0 ± 3.4	35.3 ± 4.3	40.7 ± 6.1

arterial pCO_2_, arterial partial carbondioxide pressure; arterial pO_2_, arterial partial oxygen pressure; CO, cardiac output; L-SEL-Ab, anti-L-selectin antibody; MAP, mean arterial pressure; MPAP, mean pulmonary arterial pressure; PWP, pulmonary arterial wedge pressure; PVR, peripheral vascular resistance.

**Table 3:** Tissue perfusion parameters in baboons infused with live *Escherichia coli *and treated with placebo or anti-L-selectin antibody

	Time (hours)										
	-0.5	0	1	2	4	6	10	24	32	48	72

aBE (mEQ/l)											
Placebo	2.7 ± 0.5	4.0 ± 0.6	2.4 ± 0.3	0.8 ± 0.4	-0.4 ± 0.5	-0.4 ± 0.5	-3.4 ± 0.9	-6.6 ± 0.9	-2.6 ± 1.3	3.1 ± 2.6	7.9 ± 0.6
L-SEL-Ab	4.0 ± 0.9	4.3 ± 0.9	3.1 ± 0.9	0.5 ± 1.0	-0.7 ± 1.2	-0.8 ± 1.0	-5.3 ± 1.7	-10.1 ± 2.6	-4.8 ± 2.8	2.2 ± 2.5	7.3 ± 2.1
Lactate (mmol/l)											
Placebo	3.4 ± 0.8	3.3 ± 0.8	2.4 ± 0.4	3.5 ± 0.4	5.2 ± 0.4	4.9 ± 0.4	7.3 ± 0.7	12.1 ± 1.0	7.8 ± 0.8	6.7 ± 0.8	5.2 ± 0.6
L-SEL-Ab	3.9 ± 0.9	3.9 ± 0.9	1.9 ± 0.2	4.3 ± 0.7	6.1 ± 0.9	6.1 ± 0.7	9.2 ± 1.4	14.5 ± 1.9	9.8 ± 1.0	6.7 ± 0.8	6.7 ± 1.1

aBE, arterial base excess; L-SEL-Ab, anti-L-selectin antibody.

**Table 4:** Fluid infusion requirements

	Time (hours)										
	-0.5	0	1	2	4	6	10	24	32	48	72

Ringer (ml/kg BW)											
Placebo	1 ± 0	7 ± 0	21 ± 2	40 ± 4	92 ± 4	134 ± 6	135 ± 6	159 ± 7	186 ± 11	223 ± 13	250 ± 19
L-SEL-Ab	1 ± 0	7 ± 0	20 ± 2	38 ± 4	90 ± 6	130 ± 9	131 ± 10	157 ± 11	189 ± 16	204 ± 9	219 ± 16
Hematocrit (%)											
Placebo	42.1 ± 1.3	40.3 ± 1.1	37.8 ± 1.2	35.7 ± 1.4	36.4 ± 1.6	36.6 ± 1.4	40.8 ± 1.5	35.4 ± 2.3	29.1 ± 2.0	27.5 ± 1.6	26.0 ± 2.5
L-SEL-Ab	40.7 ± 1.6	38.6 ± 1.7	37.8 ± 1.5	34.8 ± 1.3	35.5 ± 1.4	36.5 ± 1.5	40.4 ± 1.8	34.3 ± 1.5	31.4 ± 1.6	29.7 ± 1.2	28.6 ± 2.2
Protein (g/100 ml)											
Placebo	6.5 ± 0.2	6.1 ± 0.1	5.3 ± 0.2	4.5 ± 0.2	4.2 ± 0.1	4.1 ± 0.1	4.8 ± 0.1	4.6 ± 0.1	4.4 ± 0.2	4.1 ± 0.3	4.5 ± 0.4
L-SEL-Ab	6.0 ± 0.2	5.7 ± 0.2	5.0 ± 0.1	4.1 ± 0.1	4.0 ± 0.1	4.0 ± 0.2	4.5 ± 0.2	4.1 ± 0.2	4.0 ± 0.2	4.0 ± 0.3	4.4 ± 0.4

Accumulation amount of Ringer's solution over time, hematocrit and total plasma protein concentration in baboons infused with live *Escherichia coli *and treated with either placebo or anti-L-selectin antibody (L-SEL-Ab). BW = body weight.

**Table 5:** Kidney and liver function

	Time (hours)										
	-0.5	0	1	2	4	6	10	24	32	48	72

Creatinine (μmol/l)											
Placebo	86 ± 3	81 ± 5	78 ± 6	80 ± 6	93 ± 6	97 ± 11	133 ± 18	200 ± 48	189 ± 73	112 ± 10	96 ± 7
L-SEL-Ab	105 ± 6	96 ± 7	88 ± 7	93 ± 5	104 ± 6	109 ± 7	145 ± 16	240 ± 42	276 ± 65	226 ± 90^a^	121 ± 12
Urine output (ml/h)											
Placebo	nd	43.8 ± 11.0	31.9 ± 9.3	63.75 ± 29.0	111.3 ± 95.8	124.4 ± 34.3	84.4 ± 17.5	87.1 ± 27.2	80.8 ± 25.0	69.0 ± 12.6	nd
L-SEL-Ab	nd	36.88 ± 11.1	38.1 ± 12.3	66.9 ± 26.4	161.9 ± 42.0	122.5 ± 41.1	94.4 ± 25.6	79.4 ± 16.4	55.0 ± 7.2	72.0 ± 7.3	nd
BUN (mg/dl)											
Placebo	12.7 ± 1.2	12.1 ± 1.3	13.2 ± 1.3	12.2 ± 1.4	11.7 ± 1.4	11.9 ± 1.2	17.9 ± 2.0	30.8 ± 1.8	34.1 ± 4.3	38.0 ± 7.3	30.5 ± 4.3
L-SEL-Ab	13.7 ± 2.2	13.6 ± 2.2	11.6 ± 1.8	12.8 ± 2.1	11.9 ± 2.2	12.4 ± 2.0	16.4 ± 2.0	32.7 ± 2.5	39.7 ± 3.4	41.9 ± 6.3	31.6 ± 4.1
ALT (U/l)											
Placebo	12 ± 3	12 ± 2	9 ± 2	8 ± 2	25 ± 11	46 ± 20	78 ± 27	112 ± 21	93 ± 24	68 ± 12	71 ± 18
L-SEL-Ab	13 ± 2	10 ± 2	7 ± 0	7 ± 2	13 ± 4	16 ± 5	37 ± 13	93 ± 35	110 ± 28	100 ± 26	100 ± 30

Organ function in baboons infused with live *Escherichia coli *and treated with either placebo or anti-L-selectin antibody. ^a^*p* = 0.047.

ALT, alanine transferase; BUN, blood urea nitrogen; L-SEL-Ab, anti-L-selectin antibody; nd, not done.
